# Effects of Direction-Specific Training Interventions on Physical Performance and Inter-Limb Asymmetries

**DOI:** 10.3390/ijerph19031029

**Published:** 2022-01-18

**Authors:** Oliver Gonzalo-Skok, Jorge Sánchez-Sabaté, Julio Tous-Fajardo, Alberto Mendez-Villanueva, Chris Bishop, Eduardo Piedrafita

**Affiliations:** 1 Department of Return to Play, Sevilla FC, 41005 Seville, Spain; oligons@hotmail.com; 2 Faculty of Health Sciences, Universidad San Jorge, Campus Universitario, Autov A23 km 299, 50830 Villanueva de Gállego, Zaragoza, Spain; epiedrafita@usj.es; 3 INEFC Barcelona, SportsLab Performance, 08038 Barcelona, Spain; jtous17@yahoo.com; 4 ASPIRE Academy for Sports Excellence, Doha 22287, Qatar; amendezvillanueva@yahoo.com; 5 London Sport Institute, School of Science and Technology, Middlesex University, Greenlands Lane, Allianz Park, London NW4 1RL, UK; c.bishop@mdx.ac.uk

**Keywords:** resistance training, eccentric overload, functional performance, variable training

## Abstract

This study analyzed the effects of two different training programs on functional performance and inter-limb asymmetries in basketball players. Twenty-four elite youth basketball players were randomly assigned to a training program including variable unilateral horizontal movements (VUH, n = 12) or unilateral lateral movements (VUL, n = 12). Eccentric-overload training (EOT) was performed twice a week for a six-week period. Functional performance assessment included a countermovement jump test, unilateral multidirectional jumping tests (i.e., lateral, horizontal, and vertical), a rebound jump test, a limb symmetry index, a 25 m linear sprint test, and several change of direction (COD) tests. Within-group analysis showed substantial improvements in almost all functional tests in both groups (ES = 0.35–0.89). Furthermore, almost all jumping asymmetries were improved in both groups (ES = 0.38–0.69) except for vertical jumping asymmetry in VUL (ES = −0.04). Between-group analyses showed a substantial and possibly better performance in vertical jumping asymmetry and 5 m in VUH compared to that of VUL, respectively. In contrast, lateral jumping with left (ES = 1.22) and right leg (ES = 0.49) were substantially greater in VUL than in VUH. Specific force-vector training programs induced substantial improvements in both functional performance tests and inter-limb asymmetries, although greater improvements of lateral and horizontal variables may depend on the specific force vector targeted.

## 1. Introduction

Enhancing the main physical characteristics often associated with team sports (e.g., sprinting, jumping, or cutting) is a key goal of any training program with athletes [1]. Movements in team sports often require athletes to produce force unilaterally in unpredictable and variable contexts with an emphasis on eccentric and multidirectional movement patterns [2]. Lately, a growing body of literature supports the selection of exercises in different planes of motion may produce specific functional adaptations (i.e., the force vector theory) [1,3,4]. For example, studies show more beneficial effects in vertical jump height after vertical squat training [1], or a greater impact in linear sprinting performance [3] or multidirectional force application tests after either hip thrust-based training [3] or variable unilateral multidirectional training [1]. Therefore, practitioners should consider this notion as a critical aspect when programming exercises for a specific goal. Whilst such interventions have been conducted to determine the effects on measures of physical performance, far less training studies have been conducted to determine the effects on inter-limb asymmetry in team sports athletes [5,6,7,8]. Recent investigations have demonstrated that some asymmetries are indeed functional to sport performance [9], but also other studies have shown that larger asymmetries are associated with reduced physical performance [10]. The majority of studies have looked at associative analyses between asymmetry and performance, but in order to truly understand “cause and effect”, more training intervention studies are needed, because the values obtained in sporting asymmetries are realized very task specifically [11]. For example, a substantial reduction (effect size (ES) = 1.15) for inter-limb power asymmetry in unilateral squats after a six-week unilateral combined resistance training program in basketball players has been reported [6]. More recently, no meaningful changes were evident for inter-limb jumping and change of direction (COD) asymmetries after an eight-week combined strength and power training intervention in female adolescent football players [7]. Finally, Sannicandro et al. [5] showed that a six-week, twice weekly, strength, jumping, and balance training program significantly reduced existing inter-limb differences in youth tennis athletes (ES = 1.04–2.08). Despite these reductions in asymmetry, no change was evident in speed or COD speed performance thereafter. Considering the conflicting evidence about the effects of training interventions on inter-limb asymmetry, further investigations are warranted in this way.

Sport science research has shown that traditional strength training methods have been useful to improving specific movement patterns like jumping, sprinting, or cutting [3,4,6,7]. However, in recent years, the inclusion of training programs focused on eccentric overload and force vector orientation has exponentially increased in the scientific literature [1,2,8,12,13]. Eccentric-based training produces specific adaptations related to power output (e.g., an increase in muscle cross-sectional area, maximal force, and velocity of fiber shortening) [14]. It is considered that these adaptations may improve athletic performance. It should be noted that almost all eccentric overload training programs have found substantial improvements in COD performance [1,2,12]. Furthermore, jumping performance in several planes of motion has also been enhanced after such training strategies, mainly in specific-direction training [1,2,8,12]. However, only two studies have analyzed the effect of eccentric overload training on inter-limb asymmetries, both showing significant reductions in asymmetry [8,12]. Despite these positive results, there is scarce information to draw practical applications about reducing inter-limb asymmetries through the above-mentioned training strategy. Thus, the main aims of the present study were: (1) to examine the effects of two unilateral eccentric overload training strategies focused on specific force-vector training (horizontal vs. lateral) on their specific and nonspecific force vector inter-limb asymmetries and (2) to analyze the effects of these training strategies on functional performance tests in young basketball players. As the force vector theory is currently supported through several studies, we hypothesized that those training strategies performed on a specific force vector would improve the inter-limb-specific asymmetry.

## 2. Materials and Methods

### 2.1. Participants

Twenty-four young (from U-15 to U-18) highly trained basketball players (age: 16 ± 1 years; height: 193.2 ± 10.5 cm; body weight [BW]: 84.9 ± 10.8 kg) belonging to an elite basketball club volunteered to participate. Data collection took place during the competitive season’s second month, after a two-month preseason and the first month of competition. All players participated an average of ~11 h of combined training based on six basketball sessions (1 h 30′/session) and two strength/power sessions (1 h/session), added to two competitive matches (40′ of playing time/match) per week for 6 weeks. All players were competing at a national level and eight of them also at international level (i.e., European and World Basketball Championship).

**Scheme 1:**
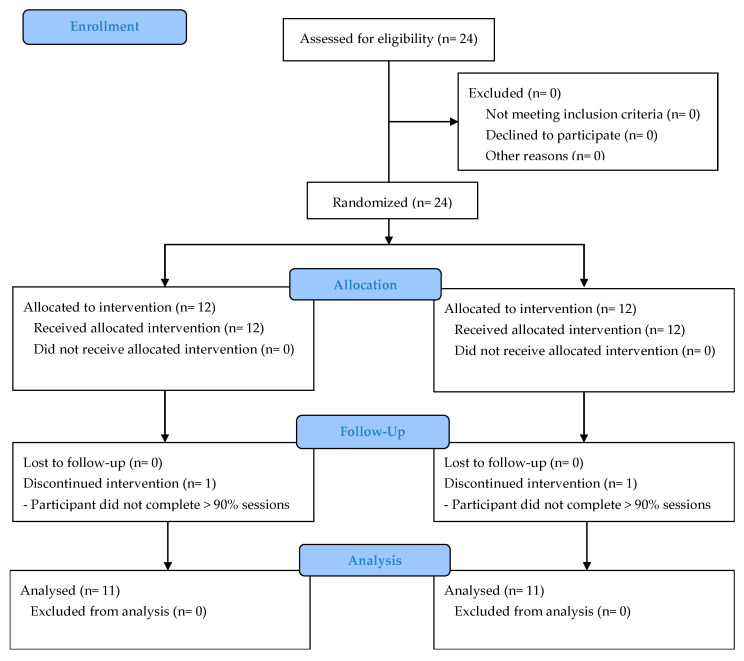
CONSORT 2010 flow diagram.

The eligibility criteria for the participants of the study were (Scheme 1): (i) to have a minimum experience of one year (range 1–4 years) in strength training, (ii) to be injury free through the intervention, (iii) to have completed >90% of the resistance training program. Informed consent was obtained from all subjects’ parents because the players were minors during the study. The current study was approved by the institutional research ethics committee and conformed to the Declaration of Helsinki recommendations.

### 2.2. Study Design

Using a controlled and randomized study design, players were divided into a variable unilateral lateral group (VUL, n = 12) or a variable unilateral horizontal group (VUH, n = 12). The training period lasted 6 weeks and was carried out in addition to the six regular basketball training sessions per week.

Tests were performed on an indoor basketball court one week before the training period and one week after the training period in the afternoon (6 p.m.–8 p.m.). These tests were realized on days with the same indoor environmental conditions (~22 °C). No meals or supplements were consumed 3 h before the tests. The assessment included, in this sequence of implementation, a bilateral countermovement jump test (CMJ), a unilateral countermovement jump test with the right leg (CMJR) and left leg (CMJL), a unilateral horizontal jump test with the right leg (HJR) and left leg (HJL), a unilateral lateral jump test with the right leg (LJR) and left leg (LJL), a repeated jumping five-second test (RJ5), a 25 m running sprint test (with 5 and 20 m split times), a 10 m sprint test (5 m + 5 m) with a 180° COD performed with the right leg (180CODR) and left leg (180CODL), a multiple-COD test (V-cut test) and a limb symmetry index (LSI) between the unilateral test. Three attempts per test were performed with 3 min of passive recovery between tests. The players were familiarized with these exercise procedures one week before the assessment by performing each test at least 6 times.

### 2.3. Procedures

#### 2.3.1. Training Intervention

**Figure 1:**
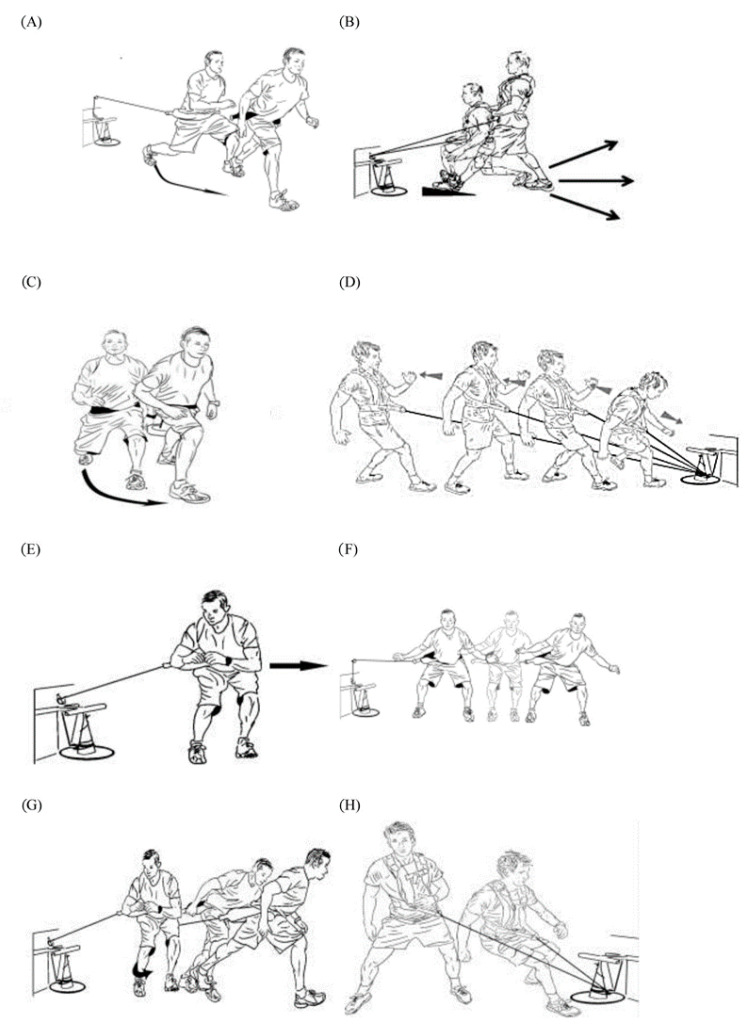
Functional eccentric overload variable unilateral horizontal training program and the corresponding force vector application: (**A**) side-step (posteroanterior/anteroposterior), (**B**) backward lunges (anteroposterior/posteroanterior), (**C**) crossover cutting (rotational/anteroposterior), and (**D**) landings and backward lunges; and functional eccentric overload variable unilateral lateral training program: (**E**) lateral squat (mediolateral/lateromedial), (**F**) defensive-like shuffling steps (mediolateral/lateromedial), (**G**) lateral crossover cutting (rotational/lateromedial), and (**H**) 90° lunge (rotational/lateromedial).

Participants performed two weekly training sessions in the afternoon (6 p.m.–8 p.m.) during a 6-week period. VUL consisted of a set of four different unilateral lateral exercises: lateral lunges, defensive shuffles, lateral crossover steps, and 90° lateral lunges with a predominant lateral force vector, whereas the VUH included a set of four different unilateral horizontal exercises: forward lunges, backward lunges, front crossover steps, and landings with backwards steps with a predominant horizontal force vector. A portable Conic Pool with 0.27 kg·m^2^ inertia and a transmission pulleys/harness setup (Versa Pulley, Costa Mesa, CA, USA) was used (Figure 1).

The training load was periodized as follows (Table 1): 6 repetitions in the 1st–2nd weeks; 8 repetitions in the 3rd–4th weeks; 10 repetitions in the 5th–6th weeks. The players were encouraged to perform the concentric phase as fast as possible, while delaying the braking action to the last third of the eccentric phase. Three minutes of passive recovery were provided between exercises. All training sessions started with the weaker leg, which was defined as the leg with the greater number in the same asymmetry direction (left or right).

#### 2.3.2. Functional Performance Tests

Every assessment (before and after) was carried out in 2 different days. All jumping tests were administered on the first day, and linear sprint and COD tests on the second day. Three attempts per test were performed with 3 min of passive recovery between tests. Unilateral tests were performed with each limb, starting with the weaker leg. First and second sessions were separated by 48 h and took place at the same time of the day (from 6 p.m. to 8 p.m.).

#### 2.3.3. Countermovement Jump Test

Lower limb vertical explosive power was assessed by an infrared optical detection system (Optojump, Microgate, Bolzano, Italy; intraclass correlation coefficient (ICC) was 0.982–0.989, and coefficient of variation (CV) was 2.7%) [2]. Variables used for analyses were CMJ, CMJR, CMJL, and both limbs’ mean (CMJpool). These tests were performed 3 times with 45 s of passive recovery between repetitions, and the best score was recorded. ICC was 0.91–0.96, and CV was 2.4–4.2%.

#### 2.3.4. Lateral and Horizontal Jump Tests

Lateral jump (LJ) and horizontal jump (HJ) performance (i.e., distance) were assessed following the Hewit et al. protocol [15]. Variables used for analyses: LJR, HJR, LJL, HJL, and both limbs’ mean (LJpool and HJpool). These tests were performed 3 times with 45 s of passive recovery between repetitions, and the best score was recorded. ICC was 0.84–0.9, and CV was 3.6–4.1%.

#### 2.3.5. Speed Tests

Running speed was evaluated on 25 m by photocell timing (Witty, Microgate, Bolzano, Italy; ICC was 0.785–0.952 and CV was 1.66–4.06%). Tests with a standing start position and with 5 m and 20 m split times were performed. Players started with the front foot 0.5 m before the starting line. The photoelectric cells were raised on tripods to 0.75 m above the ground and stood 1.5 m apart. This test was performed 3 times with 2 min of passive recovery between repetitions, and the best time was recorded. ICC was 0.79–0.83, and CV was 1.5–4.8%.

#### 2.3.6. COD Tests

**Figure 2:**
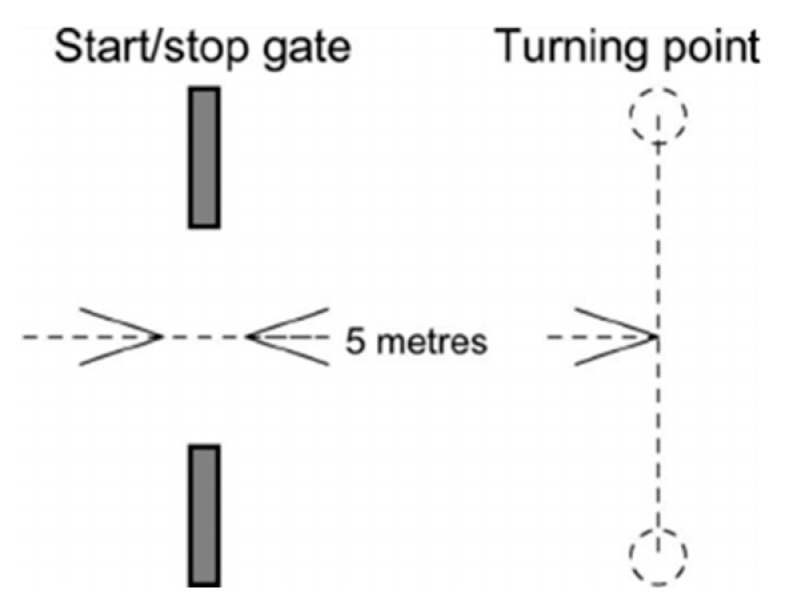
Modified 505 agility test.

A 10 m sprint test with a 180° COD was performed (Figure 2). Each player sprinted from the start/finish line, completely crossed the 5 m line with either the right or left foot, and turned 180° to sprint back to the start/finish line. Players started with the front foot 0.5 m before the starting line. The photoelectric cells were raised on tripods to 0.75 m above the ground and stood 1.5 m apart. This test was performed 3 times with 2 min of passive recovery between repetitions, and the best time was recorded. The variables used in the analyses were 180CODL and 180CODR. ICC was 0.78–0.85, and CV was 1.4–2.1%.

#### 2.3.7. V-Cut Test

Players sprinted on a 25 m zigzagging path, performing a 45°-COD every 5 m [16]. Players started with the front foot 0.5 m before the starting line. The photoelectric cells were raised on tripods to 0.75 m above the ground and stood 1.5 m apart. This test was performed 3 times with 2 min of passive recovery between repetitions, and the best time was recorded. ICC was 0.87–0.95, whereas CV was 1.2–1.7%.

#### 2.3.8. LSI

Limb symmetry index was calculated in all unilateral tests performed (LSI = worse leg/better leg × 100) [17].

### 2.4. Statistical Analyses

Data are presented as mean ± standard deviation (SD). All data were first log-transformed to reduce bias arising from non-uniformity error. The effect size (ES, 90%CI) in the selected variables was calculated using the pooled pretraining SD. Threshold values for Cohen ES statistics were >0.2 (small), >0.6 (moderate), and >1.2 (large) [18]. For intra/inter-group comparisons, the chances that the differences in performance were better/greater, similar, or worse/smaller were calculated. Quantitative chances of beneficial/better or detrimental/poorer effect were assessed qualitatively as follows: <1%, most likely not; >1–5%, very unlikely; >5–25%, unlikely; >25–75%, possible; >75–95%, likely; >95–99%, very likely; and >99%, most likely [18]. If the chance of having beneficial/better or detrimental/poorer performances was both >5%, the true difference was assessed as unclear. Otherwise, we interpreted that change as the observed chance [18].

## 3. Results

### 3.1. Participants

Only players who participated in at least 90% of the training sessions were analyzed, which resulted in two groups, 11 players/group (VUL: 16 ± 1 years, 191.2 ± 10.8 cm, 84.2 ± 10.1 kg; VUH: 16 ± 1 years, 190.1 ± 10.1 cm, 83.2 ± 9.9 kg) with no substantial anthropometric differences found at before or after the tests.

### 3.2. Intragroup Changes

Substantial improvements were found in CMJL, HJR, HJL, LSI in horizontal jumping (LSIHJ), LJL, LSI in lateral jumping (LSILJ), 180CODR, 180CODL in both groups compared to those pre-tests (Table 2 and Table 3). Furthermore, CMJR, LSI in vertical jumping (LSICMJ) and 5 m split time were also substantially enhanced in the VUH group (Table 2), whereas LJR had substantially better results in VUL (Table 3).

### 3.3. Intergroup Changes

**Figure 3:**
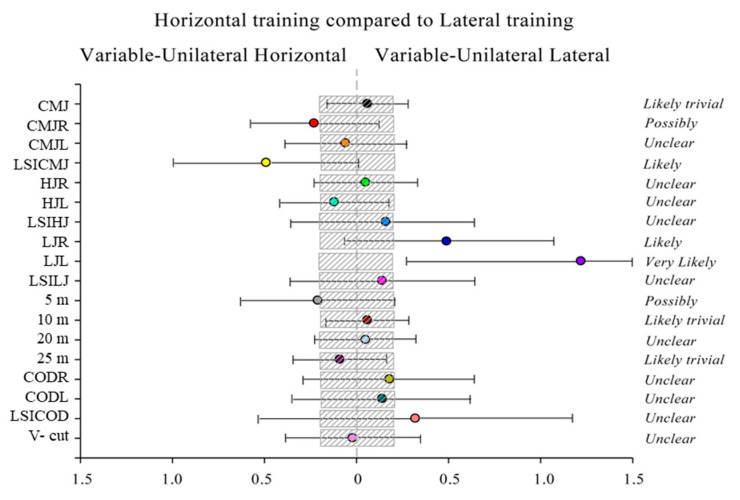
Efficiency of variable unilateral horizontal training (VUH) compared to that of the variable unilateral lateral (VUL) training program to improve bilateral countermovement jump performance (CMJ), vertical jump with the right (CMJR) and left leg (CMJJL), the between-legs asymmetry in vertical jumping (LSICMJ), horizontal jump with the right (HJR) and left leg (HJL), the between-legs asymmetry in horizontal jumping (LSIHJ), lateral jump with the right (LJR) and left leg (LJL), the between-legs asymmetry in lateral jumping (LSILJ), 5, 10, 20 and 25 m linear sprint time, a sprint of 10 m (5 + 5 m) with the right (CODR) and left leg (CODL) with a change of direction of 180°, the between-legs asymmetry in 180° change of direction (LSICOD) and a 25 m sprint with 4 changes of direction of 45° (V-cut) (the bars indicate uncertainty in the true mean changes with 90% confidence limits). Trivial areas were the smallest worthwhile change (SWC) (see Section 2).

Substantially better results were shown in LSICMJ (4.9%, 90%CI: −0.1; 9.5; 84/15/1%), in VUH in comparison to that in VUL, whereas LJR (2.8%, 90%CI: −0.4; 6.2; 81/16/2%) and LJL (5.0%, 90%CI: 1.1; 9.0; 96/3/1%) were substantially greater in VUL compared to VUH. A possibly greater performance was found in CMJR (5.1%, 90%CI: −2.4; 12.1; 57/41/2%) and 5 m split time (1.2%, 90%CI: −1.2; 3.7; 51/43/5%) in VUH compared to those of VUL (Figure 3).

## 4. Discussion

The present study compared the effects of two variable unilateral training programs emphasizing either the horizontal force component or greater lateral/rotational force vector application on inter-limb asymmetries and a battery of functional performance tests. The main findings were: (1) inter-limb asymmetry in functional jumping tests (CMJ, HJ, and LJ) substantially improved in both groups, and (2) both training programs substantially improved almost all tests, though the training adaptation principle specificity mainly prevailed, with VUH group showing greater enhancements in those tests which predominantly emphasized the horizontal (posteroanterior/anteroposterior) component (i.e., 5 m), whereas better results were found in lateral/rotational force application tests (i.e., LJR and LJL) in the VUL group.

Inter-limb asymmetries in strength and power have been considered a valid and useful tool to detect players at high risk of injury [17], to monitor a successful return to sport after an ACL injury [19], as well as to improve jump, speed, and COD performance [10,20,21]. In the present study, both groups improved almost all inter-limb jumping asymmetries (ES: from 0.38 to 0.69), therefore, these results are in line with those previously published in the literature (ES: from 0.00 to 1.15) [6,7,8,12]. However, it is interesting to note that the specificity principle was evident in the present research. Lateral jumping asymmetries’ improvement were greater in the VUL group compared to that in the VUH group (ES: 0.53 vs. 0.44). Furthermore, only one study has analyzed the effect on lateral jumping asymmetries, showing a lower ES (0.34) [12]. These differences between studies might be due to the force vector or the training strategy used (starting with the weaker leg vs. unspecified). Furthermore, horizontal jumping asymmetry was also analyzed. Despite both groups having substantially reduced such asymmetry, VUL achieved a slightly greater ES (ES: from 0.44 to 0.53). It should be noted that the horizontal force vector can be performed by posteroanterior and anteroposterior movements, in both ways. As VUL performed two posteroanterior (also lateral) exercises as well as VUH, such slight differences might be due to executing the same number of movements, in a similar way to horizontal jumping. Notwithstanding, previously published reports showed lower horizontal jumping asymmetry ES (ES: from −0.15 to 0.40) compared to that in the current study (ES: from 0.44 to 0.53) [7,8,12]. Interestingly, no study provided a beneficial effect (i.e., trivial to negative) when a horizontal force vector was not present with only one exception. Even though the exercise was not performed in the same force vector (lateral vs. horizontal), a double volume execution with the weaker leg reduced a nonspecific asymmetry, showing the importance of task-specificity and training volume.

Keeping in mind the last jumping asymmetry (i.e., vertical), trivial to moderate results (ES: from 0.08 to 0.70) have been found in previous studies [7,8,12]. While the VUH group achieved a small ES (ES: 0.38), the VUL group showed a small impairment (ES: −0.04). These differences can be justified through similar dynamic correspondence between VUH exercises and the vertical axis, but not for VUL. Thus, unless the training volume with the weaker leg is very high, where task specificity (i.e., unilateral training) might be the key factor, the specific force vector seems to be an important requisite to decrease inter-limb asymmetries, at least when training volume is similar between both limbs. Furthermore, several studies started every training session with the weaker leg [6,7,8], so it seems like those training interventions that involve unilateral strength exercises and always start with the weaker leg might be effective in reducing inter-limb asymmetries, although more studies are needed.

Considering the COD asymmetry increase, it seems that the current training strategies are not effective. However, asymmetry values are close to perfect, highlighting that total time might be a poor metric to detect existing side-to-side differences [22]. These results are in accordance with those of previous studies [7,8,12], where negative to small positive effects were found and, essentially, all players were considered as almost perfectly symmetrical. Consequently, practitioners should consider alternative test methods when looking to detect inter-limb asymmetry during CODS actions [22]. Additionally, it is important to keep in mind that COD movements are a very specific type of motor pattern which may be best served by practicing the COD task itself [23]. Therefore, it is possible that both COD assessment and training might be performed through specific motor patterns.

Neither VUH nor VUL substantially improved bilateral vertical jumping performance (ES = from 0.12 to 0.18), which is in disagreement with previous studies using other eccentric overload training (EOT) programs (ES = from 0.42 to 0.58) [1]. Furthermore, the literature concerning team sports’ players has reported considerably greater improvements in bilateral CMJ after different training strategies (i.e., resistance training [24], plyometrics [24], combined [6,25], or complex training [26]) (ES = from 0.27 to 0.71). While the vast majority of exercises performed during the above-mentioned interventions were composed by bilateral exercises executed in the axial force vector, the current intervention included only unilateral exercises executed in the horizontal or lateral plane. Thus, it is unclear whether the force vector or the bilateral–unilateral nature of the exercises might be behind those between-study differences in CMJ performance. On the other hand, unilateral vertical jumping performance was substantially improved in both groups (ES = from 0.35 to 0.62), except for CMJR (ES = 0.19) in VUL. These results are in line with those found after constant bilateral vertical training (ES = from 0.45 to 0.47) or variable unilateral multidirectional training (ES = from 0.27 to 0.39) performed with a conical pulley [1]. Thus, it seems that unilateral training performed in other force vectors (i.e., task specificity) has the potential to improve single-leg vertical jump performance.

Lateral and horizontal unilateral jumps have been moderately to largely related to linear sprinting and COD performance [27], but only two studies have analyzed the effect of EOT on lateral jumping [1,12]. Substantial improvements in LJ were achieved in both studies (ES = from 0.24 to 0.87), although possibly a greater performance was reported from multidirectional training including lateral and horizontal movements compared to those of vertical movements. Furthermore, multidirectional training showed greater ES (ES = from 0.51 to 0.87) [1] in comparison to that of a combined specific skill with handball players (ES = from 0.24 to 0.49) [12]. These results are in line with the current study (ES = from 0.33 to 0.89), with the VUL group showing stronger adaptations (Table 3, Figure 3). These results support the notion that force vector application might be a key factor to developing specific adaptations. Regarding horizontal jumping, previous training studies have reported small to moderate effects (ES = from 0.38 to 0.65). The previously observed gains in horizontal jumping performance are slightly greater than those obtained by the VUL group, and slightly smaller in comparison with those of VUH (ES = from 0.50 to 0.80) [1,28]. It may be possible that these between-study differences are due to the number of exercises addressing one force vector or the training volume carried out. However, some exercise inclusion in the VUL training program (i.e., lateral crossover step and lunge 90°), where the anteroposterior/posteroanterior force vector was also stimulated, may have contributed to intergroup difference lack in horizontal jumping.

Only the VUH group achieved a substantial improvement in 5 m, with no substantial enhancement in any other distance of linear sprinting in any group. As previously reported [1], the selected exercises focused on the first steps of the movements and thus, better results would be expected in the initial phase of a linear sprint due to the similarity of ground contact times. Thus, the ES reached in 5 m (ES = 0.50) was quite similar to the ES provided after variable unilateral multidirectional training (ES = 0.54) [1]. However, the rest of linear sprinting results (10, 20, and 25 m) are not in accordance with those found after different EOT programs (ES = from 0.10 to 0.80) [2,29,30], whereas traditional vertical-horizontal strength training programs have reported slightly lower results (ES = from 0.19 to 0.24) [31,32]. Between-study differences might be due to the training volume performed, the season moment, or the participants’ training experience/age.

These results indicate that both training programs induced substantial improvements in COD 180° performance (Table 2 and Table 3). However, the VUH group obtained better adaptations (i.e., greater mean ES) in 180°-COD tests in comparison with those in the VUL group (ES = 0.53 vs. 0.70). Paying attention to training content, there are only two studies that have included a similar EOT program [1,33]. One of them was quite similar to the current protocol (i.e., variable unilateral multidirectional) [1], and the results are within the reported ES (ES = from 0.54 to 0.61 vs. from 0.51 to 0.85) in the test that included the same number of turns (i.e., 1 COD) and distance covered (i.e., 10 m). It is possible that the 5 m improvement achieved in both groups at post-test related to a possible eccentric overload in the forward lunge (similar biomechanics to 180°-COD braking in the same position) might be responsible for prompting these enhancements and the between-group differences in the 180°-COD ES mean (i.e., VUH vs. VUL), as there were possibly and substantial differences in 5 m, respectively. On the other hand, the V-cut test was not substantially improved in any group, which is in agreement with a study that included a similar COD test (4 × 100° cut angles along 20 m) after a six-week training program performing from five to eight sets of a single horizontal exercise (front step) on the CP [33]. A priori, we considered this test more related to lateral/torsional movements than horizontal movements. Indeed, substantial gains in the V-cut test (ES = 1.22) were provided after a combined lateral/rotational EOT + vibrations in young soccer players [2]. It is worth noting that the main determinants in COD ability are linear sprinting, eccentric strength, technique, and anthropometry [34]. As only the VUH group improved one of these components (5 m), no eccentric overload was achieved in the lateral squat, and considering that eccentric overload in this exercise was not noted in our pilot studies, it seems that the repeated COD ability improvement might be based on the development of at least an eccentric overload in the lateral squat exercise, just as it was hypothesized in the above-mentioned study where the YoYo Squat was used.

The new approach based on performing exclusively one set per exercise seems to be time-effective to improve the most important abilities in basketball players. Indeed, strength and conditioning coaches could choose the required specific force vector on the selected ability by position on the court and role during the game. Furthermore, unilateral exercises might help balance both limbs and achieve the two-fold aim in a time-efficient manner: improve performance and minimize the risk of injury. When the aim is to decrease inter-limb asymmetry, both the specific force vector starting with the weaker leg and task-specificity seems to be the appropriated strategy when the volume is equated between both limbs. Finally, in the practical context, our proposal is fine-tuning permanently (i.e., inter-, and intra-set and session) and not to choose one or the other option. The idea is to evolve the progression by fine-tuning vectors, external elements, perturbations, and unilateral/bilateral exercises. All depends on what will be expected based on specific player demands.

This work has some limitations that should be acknowledged. Firstly, the body composition was not analyzed, considering some studies have shown substantial improvements in muscle volume and positive changes in body composition after eccentric overload training [35,36]. This type of adaptation could be relevant to athletes’ performance. Secondly, the sample of the study was elite youth basketball players, so it was not possible to have a control group. Moreover, the restricted statistical power because of the sample size in this study may have influenced the significance of some of the statistical comparisons conducted. A post hoc power analysis revealed that, for the lowest effect size of interest observed in the present study (d = 0.3), the number of players would have been approximately 137 in each group to obtain statistical power at the recommended 0.80 level. Future research should control these variables.

## 5. Conclusions

A specific force vector training program induced substantial improvements in both specific and nonspecific inter-limb asymmetries and functional performance tests, although greater improvements of lateral and horizontal variables may depend on the specific force vector targeted. Therefore, the force vector application (i.e., anteroposterior/posteroanterior vs. lateral/torsional) and task specificity may play an important role in developing different and specific functional adaptations.

## Figures and Tables

**Table 1:** Program training exercises and load.

Week	Session	VUH Exercises	VUL Exercises	Sets × Repetitions
1	1–2	Side-step	Lateral squat	1 × 6
		Backward lunges	Defensive-like shuffling steps	1 × 6
		Crossover cutting	Lateral crossover cutting	1 × 6
		Landing and backward lunges	90° lunge	1 × 6
2	3–4	Side-step	Lateral squat	1 × 6
		Backward lunges	Defensive-like shuffling steps	1 × 6
		Crossover cutting	Lateral crossover cutting	1 × 6
		Landing and backward lunges	90° lunge	1 × 6
3	5–6	Side-step	Lateral squat	1 × 8
		Backward lunges	Defensive-like shuffling steps	1 × 8
		Crossover cutting	Lateral crossover cutting	1 × 8
		Landing and backward lunges	90° lunge	1 × 8
4	7–8	Side-step	Lateral squat	1 × 8
		Backward lunges	Defensive-like shuffling steps	1 × 8
		Crossover cutting	Lateral crossover cutting	1 × 8
		Landing and backward lunges	90° lunge	1 × 8
5	9–10	Side-step	Lateral squat	1 × 10
		Backward lunges	Defensive-like shuffling steps	1 × 10
		Crossover cutting	Lateral crossover cutting	1 × 10
		Landing and backward lunges	90° lunge	1 × 10
6	11–12	Side-step	Lateral squat	1 × 10
		Backward lunges	Defensive-like shuffling steps	1 × 10
		Crossover cutting	Lateral crossover cutting	1 × 10
		Landing and backward lunges	90° lunge	1 × 10

**Table 2:** Changes in performance after variable unilateral horizontal (VUH, n = 11) eccentric overload training.

	Pre-Test	Post-Test	% (CL90%)	ES (CL90%)	Chances	Outcome
CMJ (cm)	35.4 ± 5.2	36.1 ± 5.3	1.9 (−0.8; 4.6)	0.12 (−0.05; 0.28)	19/81/0%	Likely trivial
CMJR (cm)	17.5 ± 3.0	19.2 ± 2.5	10.4 (2.7; 18.8)	0.51 (0.13; 0.88)	92/8/0%	Likely
CMJL (cm)	19.0 ± 2.4	20.6 ± 2.2	8.6 (3.5; 13.9)	0.62 (0.26; 0.99)	97/3/0%	Very Likely
LSICMJ (%)	88.8 ± 9.3	92.6 ± 4.6	4.7 (0.0; 9.7)	0.38 (0.00; 0.77)	80/19/1%	Likely
HJR (cm)	161.0 ± 16.5	169.8 ± 15.5	5.6 (2.5; 8.7)	0.50 (0.22; 0.77)	96/4/0%	Very Likely
HJL (cm)	157.0 ± 14.5	169.5 ± 16.5	7.9 (5.0; 10.8)	0.80 (0.52; 1.08)	100/0/0%	Most Likely
LSIHJ (%)	94.7 ± 4.5	97.0 ± 2.5	2.5 (0.0; 5.0)	0.46 (0.01; 0.91)	84/15/1%	Likely
LJR (cm)	149.5 ± 13.7	154.4 ± 11.4	3.4 (−0.1; 6.9)	0.33 (−0.01; 0.67)	73/26/1%	Possibly
LJL (cm)	149.7 ± 8.1	157.4 ± 10.7	5.0 (3.1; 7.0)	0.83 (0.51; 1.15)	100/0/0%	Most Likely
LSILJ (%)	92.5 ± 5.4	95.2 ± 3.8	2.9 (1.0; 4.9)	0.44 (0.15; 0.73)	92/8/0%	Likely
5 m (s)	1.06 ± 0.06	1.03 ± 0.06	2.9 (1.7; 4.1)	0.50 (0.28; 0.72)	98/2/0%	Very Likely
10 m (s)	1.81 ± 0.07	1.79 ± 0.08	0.9 (0.2; 1.5)	0.20 (0.04; 0.35)	48/52/0%	Possibly
20 m (s)	3.11 ± 0.13	3.11 ± 0.13	0.0 (−0.8; 0.8)	0.01 (−0.18; 0.19)	4/92/3%	Likely trivial
25 m (s)	3.73 ± 0.15	3.74 ± 0.15	−0.1 (−0.5; 0.3)	−0.03 (−0.13; 0.07)	0/99/1%	Very Likely trivial
180°-CODR (s)	2.69 ± 0.09	2.63 ± 0.10	2.0 (0.8; 3.3)	0.55 (0.21; 0.90)	95/4/0%	Very Likely
180°-CODL (s)	2.71 ± 0.08	2.64 ± 0.11	2.6 (1.1; 4.1)	0.85 (0.35; 1.35)	98/2/0%	Very Likely
LSICOD (%)	98.1 ± 1.2	97.1 ± 2.2	−1.0 (−2.3; 0.4)	−0.76 (−1.79; 0.27)	6/11/83%	Likely harmful
V-cut (s)	6.91 ± 0.29	6.82 ± 0.19	1.2 (0.0; 2.3)	0.25 (0.00; 0.51)	65/35/0%	Possibly

Note: CMJ: countermovement jump; CMJR and CMJL: countermovement jump with right and left leg; LSICMJ: limb symmetry index in CMJ; HJR and HJL: horizontal jump with right and left leg; LSIHJ: limb symmetry index in HJ; LJR and LJL: lateral jump with right and left leg; LSILJ: limb symmetry index in LJ; 180°-CODR and 180°-CODL: 5 + 5 m sprint with 180° change of direction with right and left leg; LSICOD: limb symmetry index in COD; CL: confidence limit; ES: effect size.

**Table 3:** Changes in performance after variable unilateral lateral (VUL, n = 11) eccentric overload training.

	Pre-Test	Post-Test	% (CL90%)	ES (CL90%)	Chances	Outcome
CMJ (cm)	34.9 ± 5.0	35.8 ± 4.6	2.8 (0.8; 4.9)	0.18 (0.05; 0.30)	37/63/0%	Possibly
CMJR (cm)	18.6 ± 4.1	19.5 ± 4.3	4.8 (2.1; 7.6)	0.19 (0.08; 0.29)	42/58/0%	Possibly
CMJL (cm)	19.0 ± 3.7	20.3 ± 3.3	7.5 (3.6; 11.5)	0.35 (0.17; 0.52)	92/8/0%	Likely
LSICMJ (%)	91.1 ± 6.9	90.9 ± 7.8	−0.3 (−2.8; 2.1)	−0.04 (−0.34; 0.25)	8/74/18%	Unclear
HJR (cm)	165.7 ± 21.1	175.6 ± 19.5	6.2 (4.0; 8.2)	0.44 (0.29; 0.59)	99/1/0%	Very Likely
HJL (cm)	166.8 ± 20.8	177.0 ± 19.4	6.3 (3.6; 9.1)	0.43 (0.25; 0.61)	98/2/0%	Very Likely
LSIHJ (%)	94.0 ± 4.1	97.1 ± 2.7	3.3 (1.6; 4.9)	0.69 (0.35; 1.04)	99/1/0%	Very Likely
LJR (cm)	146.1 ± 22.4	156.9 ± 17.9	8.0 (4.8; 11.2)	0.46 (0.28; 0.64)	99/1/0%	Very Likely
LJL (cm)	143.2 ± 15.0	158.5 ± 18.2	10.6 (7.2; 14.1)	0.89 (0.62; 1.17)	100/0/0%	Almost Certain
LSILJ (%)	92.5 ± 5.9	96.0 ± 4.3	3.9 (1.0; 6.8)	0.53 (0.13; 0.92)	92/8/0%	Likely
5 m (s)	1.06 ± 0.06	1.04 ± 0.05	1.7 (−0.4; 3.8)	0.29 (−0.07; 0.64)	66/32/2%	Possibly
10 m (s)	1.82 ± 0.07	1.80 ± 0.07	1.1 (0.5; 1.7)	0.27 (0.11; 0.43)	68/25/7%	Possibly
20 m (s)	3.12 ± 0.11	3.12 ± 012	0.1 (−0.7; 1.0)	0.04 (−0.18; 0.26)	11/85/4%	Likely trivial
25 m (s)	3.74 ± 0.13	3.76 ± 0.15	−0.5 (−1.4; 0.4)	−0.12 (−0.35; 0.10)	1/71/27%	Possibly trivial
180°-CODR (s)	2.67 ± 0.13	2.59 ± 0.17	2.8 (1.2; 4.8)	0.56 (0.24; 0.88)	97/3/0%	Very Likely
180°-CODL (s)	2.71 ± 0.17	2.62 ± 0.16	3.3 (1.3; 5.2)	0.51 (0.20; 0.82)	95/5/0%	Very Likely
LSICOD (%)	97.1 ± 2.5	96.8 ± 2.0	−0.3 (−1.7; 1.2)	−0.09 (−0.63; 0.44)	17/47/36%	Possibly harmful
V-cut (s)	6.99 ± 0.36	6.91 ± 0.32	1.1 (−0.5; 2.6)	0.20 (−0.08; 0.48)	49/49/1%	Possibly

Note: CMJ: countermovement jump; CMJR and CMJL: countermovement jump with right and left leg; LSICMJ: limb symmetry index in CMJ; HJR and HJL: horizontal jump with right and left leg; LSIHJ: limb symmetry index in HJ; LJR and LJL: lateral jump with right and left leg; LSILJ: limb symmetry index in LJ; 180°-CODR and 180°-CODL: 5 + 5 m sprint with 180° change of direction with right and left leg; LSICOD: limb symmetry index in COD; CL: confidence limit; ES: effect size.

## Data Availability

The datasets generated and analyzed for this study can be requested by correspondence from the authors at osanchez@usj.es.

## References

[1] Gonzalo-Skok O., Tous-Fajardo J., Valero-Campo C., Berzosa C., Bataller A.V., Arjol-Serrano J.L., Moras G., Mendez-Villanueva A. (2017). Eccentric-Overload Training in Team-Sport Functional Performance: Constant Bilateral Vertical Versus Variable Unilateral Multidirectional Movements. Int. J. Sports Physiol. Perform..

[2] Tous-Fajardo J., Gonzalo-Skok O., Arjol-Serrano J.L., Tesch P. (2016). Enhancing Change-of-Direction Speed in Soccer Players by Functional Inertial Eccentric Overload and Vibration Training. Int. J. Sports Physiol. Perform..

[3] Contreras B., Vigotsky A.D., Schoenfeld B.J., Beardsley C., McMaster D.T., Reyneke J.H.T., Cronin J.B. (2017). Effects of a Six-Week Hip Thrust vs. Front Squat Resistance Training Program on Performance in Adolescent Males: A Randomized Controlled Trial. J. Strength Cond. Res..

[4] Abade E., Silva N., Ferreira R., Baptista J., Gonçalves B., Osório S., Viana J. (2021). Effects of Adding Vertical or Horizontal Force-Vector Exercises to In-Season General Strength Training on Jumping and Sprinting Performance of Youth Football Players. J. Strength Cond. Res..

[5] Sannicandro I., Cofano G., Rosa R.A., Piccinno A. (2014). Balance Training Exercises Decrease Lower-Limb Strength Asymmetry in Young Tennis Players. J. Sports Sci. Med..

[6] Gonzalo-Skok O., Tous-Fajardo J., Suarez-Arrones L., Arjol-Serrano J.L., Casajús J.A., Mendez-Villanueva A. (2017). Single-Leg Power Output and Between-Limbs Imbalances in Team-Sport Players: Unilateral Versus Bilateral Combined Resistance Training. Int. J. Sports Physiol. Perform..

[7] Pardos-Mainer E., Casajús J.A., Bishop C., Gonzalo-Skok O. (2020). Effects of Combined Strength and Power Training on Physical Performance and Interlimb Asymmetries in Adolescent Female Soccer Players. Int. J. Sports Physiol. Perform..

[8] Gonzalo-Skok O., Moreno-Azze A., Arjol-Serrano J.L., Tous-Fajardo J., Bishop C. (2019). A Comparison of 3 Different Unilateral Strength Training Strategies to Enhance Jumping Performance and Decrease Interlimb Asymmetries in Soccer Players. Int. J. Sports Physiol. Perform..

[9] Bini R.R., Hume P.A. (2015). Relationship between Pedal Force Asymmetry and Performance in Cycling Time Trial. J. Sports Med. Phys. Fitness.

[10] Bishop C., Turner A., Read P. (2018). Effects of Inter-Limb Asymmetries on Physical and Sports Performance: A Systematic Review. J. Sports Sci..

[11] Maloney S.J. (2019). The Relationship Between Asymmetry and Athletic Performance: A Critical Review. J. Strength Cond. Res..

[12] Madruga-Parera M., Bishop C., Fort-Vanmeerhaeghe A., Beato M., Gonzalo-Skok O., Romero-Rodríguez D. (2020). Effects of 8 Weeks of Isoinertial vs. Cable-Resistance Training on Motor Skills Performance and Interlimb Asymmetries. J. Strength Cond. Res..

[13] Gonzalo-Skok O., Sánchez-Sabaté J., Izquierdo-Lupón L., Sáez de Villarreal E. (2019). Influence of Force-Vector and Force Application Plyometric Training in Young Elite Basketball Players. Eur. J. Sport Sci..

[14] Suchomel T.J., Wagle J.P., Douglas J., Taber C.B., Harden M., Haff G.G., Stone M.H. (2019). Implementing Eccentric Resistance Training-Part 1: A Brief Review of Existing Methods. J. Funct. Morphol. Kinesiol..

[15] Hewit J.K., Cronin J.B., Hume P.A. (2012). Asymmetry in Multi-Directional Jumping Tasks. Phys. Ther. Sport Off. J. Assoc. Chart. Physiother. Sports Med..

[16] Gonzalo-Skok O., Tous-Fajardo J., Suarez-Arrones L., Arjol-Serrano J.L., Casajús J.A., Mendez-Villanueva A. (2015). Validity of the V-Cut Test for Young Basketball Players. Int. J. Sports Med..

[17] Gustavsson A., Neeter C., Thomeé P., Silbernagel K.G., Augustsson J., Thomeé R., Karlsson J. (2006). A Test Battery for Evaluating Hop Performance in Patients with an ACL Injury and Patients Who Have Undergone ACL Reconstruction. Knee Surg. Sports Traumatol. Arthrosc. Off. J. ESSKA.

[18] Hopkins W.G., Marshall S.W., Batterham A.M., Hanin J. (2009). Progressive Statistics for Studies in Sports Medicine and Exercise Science. Med. Sci. Sports Exerc..

[19] Ardern C.L., Webster K.E., Taylor N.F., Feller J.A. (2011). Return to the Preinjury Level of Competitive Sport after Anterior Cruciate Ligament Reconstruction Surgery: Two-Thirds of Patients Have Not Returned by 12 Months after Surgery. Am. J. Sports Med..

[20] Fort-Vanmeerhaeghe A., Bishop C., Buscà B., Aguilera-Castells J., Vicens-Bordas J., Gonzalo-Skok O. (2020). Inter-Limb Asymmetries Are Associated with Decrements in Physical Performance in Youth Elite Team Sports Athletes. PLoS ONE.

[21] Bishop C., Brashill C., Abbott W., Read P., Lake J., Turner A. (2021). Jumping Asymmetries Are Associated With Speed, Change of Direction Speed, and Jump Performance in Elite Academy Soccer Players. J. Strength Cond. Res..

[22] Madruga-Parera M., Romero-Rodríguez D., Bishop C., Beltran-Valls M.R., Latinjak A.T., Beato M., Fort-Vanmeerhaeghe A. (2019). Effects of Maturation on Lower Limb Neuromuscular Asymmetries in Elite Youth Tennis Players. Sports.

[23] Dos’Santos T., McBurnie A., Comfort P., Jones P.A. (2019). The Effects of Six-Weeks Change of Direction Speed and Technique Modification Training on Cutting Performance and Movement Quality in Male Youth Soccer Players. Sports.

[24] de Hoyo M., Gonzalo-Skok O., Sañudo B., Carrascal C., Plaza-Armas J.R., Camacho-Candil F., Otero-Esquina C. (2016). Comparative Effects of In-Season Full-Back Squat, Resisted Sprint Training, and Plyometric Training on Explosive Performance in U-19 Elite Soccer Players. J. Strength Cond. Res..

[25] Rodríguez-Rosell D., Franco-Márquez F., Pareja-Blanco F., Mora-Custodio R., Yáñez-García J.M., González-Suárez J.M., González-Badillo J.J. (2016). Effects of 6 Weeks Resistance Training Combined With Plyometric and Speed Exercises on Physical Performance of Pre-Peak-Height-Velocity Soccer Players. Int. J. Sports Physiol. Perform..

[26] Freitas T.T., Martinez-Rodriguez A., Calleja-González J., Alcaraz P.E. (2017). Short-Term Adaptations Following Complex Training in Team-Sports: A Meta-Analysis. PLoS ONE.

[27] Meylan C., McMaster T., Cronin J., Mohammad N.I., Rogers C., Deklerk M. (2009). Single-Leg Lateral, Horizontal, and Vertical Jump Assessment: Reliability, Interrelationships, and Ability to Predict Sprint and Change-of-Direction Performance. J. Strength Cond. Res..

[28] Gonzalo-Skok O., Tous-Fajardo J., Arjol-Serrano J.L., Suarez-Arrones L., Casajús J.A., Mendez-Villanueva A. (2016). Improvement of Repeated-Sprint Ability and Horizontal-Jumping Performance in Elite Young Basketball Players With Low-Volume Repeated-Maximal-Power Training. Int. J. Sports Physiol. Perform..

[29] Askling C., Karlsson J., Thorstensson A. (2003). Hamstring Injury Occurrence in Elite Soccer Players after Preseason Strength Training with Eccentric Overload. Scand. J. Med. Sci. Sports.

[30] de Hoyo M., de la Torre A., Pradas F., Sañudo B., Carrasco L., Mateo-Cortes J., Domínguez-Cobo S., Fernandes O., Gonzalo-Skok O. (2015). Effects of Eccentric Overload Bout on Change of Direction and Performance in Soccer Players. Int. J. Sports Med..

[31] Los Arcos A., Yanci J., Mendiguchia J., Salinero J.J., Brughelli M., Castagna C. (2014). Short-Term Training Effects of Vertically and Horizontally Oriented Exercises on Neuromuscular Performance in Professional Soccer Players. Int. J. Sports Physiol. Perform..

[32] López-Segovia M., Palao Andrés J.M., González-Badillo J.J. (2010). Effect of 4 Months of Training on Aerobic Power, Strength, and Acceleration in Two under-19 Soccer Teams. J. Strength Cond. Res..

[33] de Hoyo M., Sañudo B., Carrasco L., Domínguez-Cobo S., Mateo-Cortes J., Cadenas-Sánchez M.M., Nimphius S. (2015). Effects of Traditional Versus Horizontal Inertial Flywheel Power Training on Common Sport-Related Tasks. J. Hum. Kinet..

[34] Brughelli M., Cronin J., Levin G., Chaouachi A. (2008). Understanding Change of Direction Ability in Sport: A Review of Resistance Training Studies. Sports Med..

[35] Núñez F.J., Santalla A., Carrasquila I., Asian J.A., Reina J.I., Suarez-Arrones L.J. (2018). The Effects of Unilateral and Bilateral Eccentric Overload Training on Hypertrophy, Muscle Power and COD Performance, and Its Determinants, in Team Sport Players. PLoS ONE.

[36] Suarez-Arrones L., Saez de Villarreal E., Núñez F.J., Di Salvo V., Petri C., Buccolini A., Maldonado R.A., Torreno N., Mendez-Villanueva A. (2018). In-Season Eccentric-Overload Training in Elite Soccer Players: Effects on Body Composition, Strength and Sprint Performance. PLoS ONE.

